# Structural equation modeling reveals proline as the dominant pathway governing rice seed germination under chilling stress

**DOI:** 10.3389/fpls.2026.1889530

**Published:** 2026-07-13

**Authors:** Siyu Cheng, Haonan Luo, Zhihao Chen, Zhilei Liu, Pengfei Li, Cailian Yu, Xingjian Xu, Li Wen, Xianlong Peng

**Affiliations:** 1 College of Resources and Environment, Northeast Agricultural University, Harbin, China; 2 National Key Laboratory of Smart Farm Technologies and Systems, Harbin, China; 3 Key Laboratory of Germplasm Enhancement, Physiology and Ecology of Food Crops in Cold Region, Ministry of Education, Northeast Agricultural University, Harbin, China; 4 The School of Material Science and Chemical Engineering, Harbin University of Science and Technology, Harbin, China; 5 Hinggan League Academy of Agricultural and Animal, Ulanhot, China; 6 Husbandry Sciences, Inner Mongolia Key Laboratory of Rice Breeding Innovation in Northern Cold Regions, Ulanhot, China

**Keywords:** chilling stress, direct-seeded rice, prohexadione-calcium, seed germination, selenium

## Abstract

**Background:**

Low-temperature stress limits the germination of direct-seeded rice (Oryza sativa L.), yet the physiological mechanisms underlying antioxidant defense and reserve mobilization remain unclear. Prohexadione-calcium (PC) modulates gibberellin biosynthesis and oxidative metabolism, while selenium (Se) functions as an antioxidant cofactor; however, their combined effects on cold-stressed germination in rice have not been systematically investigated in terms of integrated physiological regulation involving antioxidant and metabolic pathways.

**Methods:**

Two rice varieties with contrasting cold sensitivity (Hajingdao 10, sensitive; Longjing 31, tolerant) were subjected to seed soaking treatments: water (control), selenium (Se, 0.3 mg L^-^¹), prohexadione-calcium (PC, 0.1 mg L^-^¹), PC+Se (0.1 + 0.3 mg L^-^¹), and Se priming. Germination rate, emergence, yield, and physiological indices (antioxidant enzymes, proline, MDA, amylase, soluble protein) were measured under controlled and field conditions. Selenium priming (SP) showed a comparatively weaker improvement in germination and physiological traits than Se soaking and PC+Se treatment. Structural equation modeling (SEM) identified key physiological drivers of germination.

**Results:**

The combined PC+Se treatment significantly enhanced germination rate and seedling emergence compared with controls. SEM revealed that proline accumulation exerted a strong positive direct effect on germination rate, whereas α-amylase activity showed a significant negative effect. In contrast, SOD and POD did not show significant direct effects on germination rate, but contributed indirectly through modulation of oxidative stress and lipid peroxidation (MDA). No significant changes in gibberellin or abscisic acid were observed.

**Conclusion:**

Low-temperature germination in rice is primarily driven by osmotic regulation, with proline accumulation acting as the key positive determinant, while carbohydrate mobilization (α-amylase activity) serves as a stress-sensitive limiting factor. Antioxidant enzyme responses contribute mainly as upstream physiological buffers rather than direct determinants of germination performance. PC+Se seed soaking is an effective strategy for improving early seedling establishment under chilling conditions.

## 1 Introduction

Seed germination represents a sensitive developmental stage in the life cycle of rice (*Oryza sativa* L.), during which low temperature often becomes a primary constraint limiting successful seedling establishment. In China, direct-seeded rice has become increasingly common in recent years, largely driven by rising labor costs and declining rice purchase prices. At present, the rice planting area exceeds approximately 29 million hectares, positioning China among the leading rice-producing countries worldwide (FAO, 2023; USDA FAS, 2025). Nevertheless, the production system is frequently challenged by unstable climatic conditions. Early spring low-temperature events associated with the El Niño–Southern Oscillation (ENSO) are common in northern rice-growing regions and are closely linked to poor germination performance and yield reduction (IPCC, 2021; NOAA Climate Prediction Center, 2024; Yu et al., 2024; Pan et al., 2024; Qi et al., 2023).

Approaches aimed at improving cold tolerance, particularly through cultivar selection and seed-based treatments, have proven effective in alleviating chilling injury. Seed soaking, priming, and coating with salts, amino acids, or plant growth regulators have been widely reported to enhance germination under low-temperature stress (Paparella et al., 2015; Zhang et al., 2023). Among these approaches, selenium (Se) and calcium-based regulators such as prohexadione-calcium (PC) have received increasing attention due to their roles in stress adaptation and antioxidant regulation. However, their combined influence on rice germination under chilling conditions has not yet been systematically investigated (Suo et al., 2024; Hussain et al., 2023; Li et al., 2023; Zhang et al., 2023).

Prohexadione-calcium (PC) regulates plant growth by inhibiting key steps in gibberellin biosynthesis, particularly the late stages of the GA metabolic pathway, and is also associated with the regulation of oxidative processes in plants (Rademacher, 2015). Although it is mainly used to control plant height and reduce lodging, recent studies suggest that PC may enhance abiotic stress tolerance through improved reactive oxygen species (ROS) homeostasis and reduced oxidative injury (Li et al., 2023). Under low-temperature stress, excessive ROS accumulation disrupts membrane stability and interferes with cellular metabolism, thereby inhibiting seed germination. In this context, PC may help maintain cellular integrity by modulating oxidative processes and contributing to cellular stress tolerance.

Selenium (Se) is recognized as a beneficial element for plants under environmental stress conditions (Hussain et al., 2023). At appropriate concentrations, Se enhances the activities of antioxidant enzymes such as superoxide dismutase (SOD), peroxidase (POD), and catalase (CAT), thereby reducing ROS accumulation and limiting lipid peroxidation (Suo et al., 2024). It is also associated with improved osmotic regulation and membrane stability under stress environments. During germination, these effects may contribute to more efficient reserve utilization and improved metabolic activity, ultimately supporting early seedling growth. Although Se has been shown to enhance germination and stress tolerance under low temperature, drought, and salinity stress, its integrated physiological roles in rice under chilling conditions remain insufficiently understood, particularly regarding the relative contributions of antioxidant defense, osmotic regulation, and reserve metabolism.

Despite individual evidence for PC and Se in cold tolerance, their combined application as a seed soaking treatment for direct-seeded rice under chilling stress has not been studied. Specifically, the relative contributions of antioxidant defense, reserve mobilization, osmotic adjustment, and hormone regulation to low-temperature germination have not been quantified in an integrated analytical framework. Understanding these mechanistic priorities is essential for optimizing seed treatment strategies.

Seed germination under chilling stress is governed by a coordinated network involving antioxidant defense, osmotic adjustment, reserve mobilization, and hormonal signaling. These processes are tightly interconnected, making it difficult to separate their individual contributions using single-factor approaches. Previous studies have mainly focused on individual physiological traits such as antioxidant enzyme activity, endogenous hormone levels, or starch degradation, whereas their relative importance and interactions under low-temperature conditions remain unclear. Structural equation modeling (SEM) provides an effective approach to quantify these relationships and identify the key pathways driving germination performance. This study therefore aimed to: (1) evaluate the effects of PC and Se seed soaking, individually and in combination, on germination performance of two rice varieties with contrasting cold sensitivity; (2) quantify the relative contributions of key physiological pathways to germination rate using structural equation modeling; (3) assess whether hormonal regulation (ABA/GA balance) or antioxidant defense constitutes the primary determinant of cold tolerance; and (4) validate the agronomic effectiveness of the optimal treatment in pot and field experiments.

## 2 Materials and methods

### 2.1 Test materials

**Figure 1:**
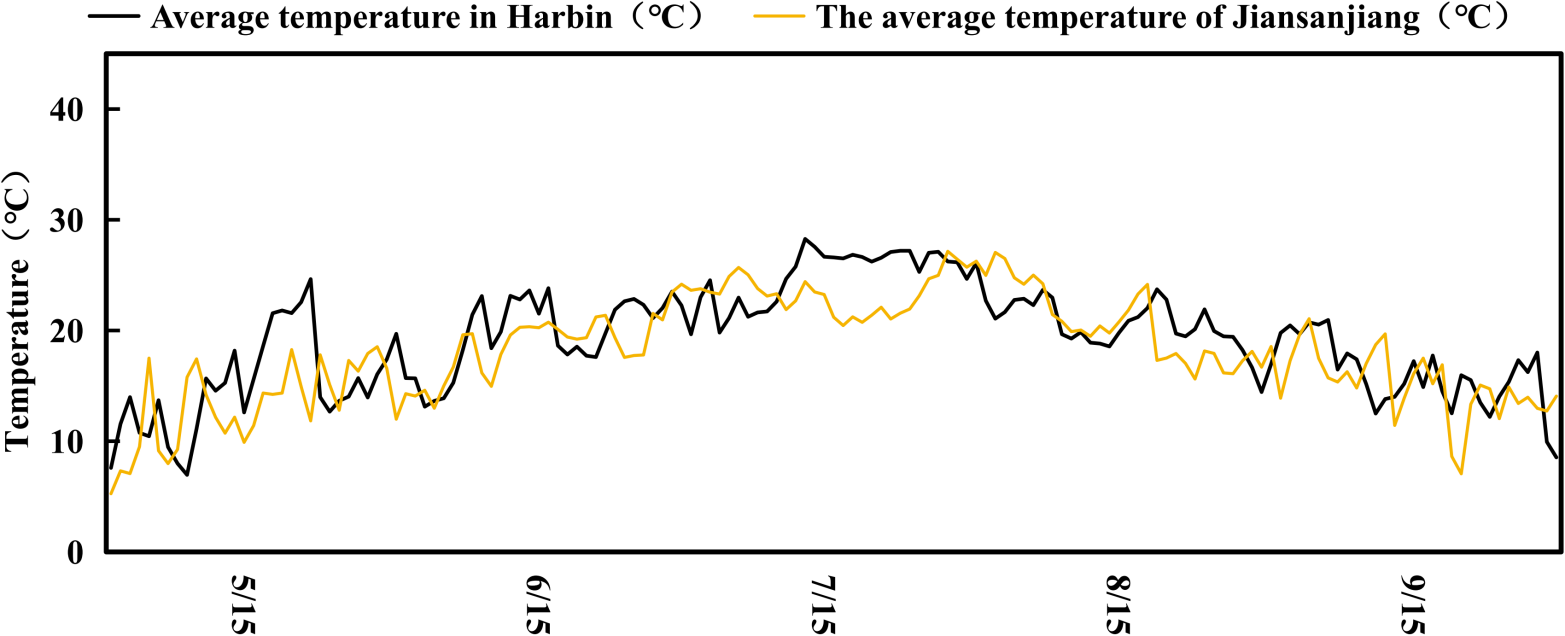
Average daily temperature (°C) during the experimental period in Harbin and Jiansanjiang.

Hajingdao 10 (low-temperature–sensitive) and Longjing 31 (low-temperature–resistant) of *Oryza sativa* L. were used as experimental materials. The seeds were screened using an ammonium sulfate solution (specific gravity = 1.08) to remove unfilled seeds and then naturally dried for use. The soil used for the pot experiment was collected from Mollisol paddy soil (127.15595°E, 44.91417°N) in the Wuchang area, Harbin, Heilongjiang Province, China, classified to the great group level as Aquic Hapludolls. Field experiments were carried out at Harbin, Qianfeng Farm, and Erdaohe Farm. The basic fertility of the tested soil is shown in **Table 1**, and the test temperature is shown in **Figure 1**.

**Table 1:** Baseline soil chemical properties of the experimental sites.

Site	pH	Organic matter	Alkaline N	Available P	Available K
		(g/kg)	(mg/kg)	(mg/kg)	(mg/kg)
Wuchang (pot)	6.17	39	168	69.8	97.1
Harbin 2021(field)	7.32	38	129	15.4	141
Erdaohe 2022 (field)	5.71	43	163	23	175
Qianfeng 2022 (field)	5.24	43	153	28	157

### 2.2 Experimental design

#### 2.2.1 Germination test

A two-factor experimental design involving two rice varieties (Hajingdao 10 and Longjing 31) was employed. Five treatments with four replicates were applied to each cultivar, and each replicate consisted of 100 seeds. The treatments included water-soaked seeds (W), 0.3 mg L^-^¹ selenium (Se)-soaked seeds, 0.1 mg L^-^¹ prohexadione-calcium (PC)-soaked seeds, combined PC + Se treatment (0.1 + 0.3 mg L^-^¹), and a selenium priming (SP) treatment. The concentrations of Se (0.3 mg L^-^¹) and PC (0.1 mg L^-^¹) were selected based on preliminary dose–response experiments, in which multiple concentration gradients were evaluated and the levels showing the most consistent improvement in germination performance under chilling stress were chosen. These concentrations were also within the effective ranges reported in previous studies on seed priming and abiotic stress tolerance. The Se priming (SP) treatment was included as a reference priming method commonly used in seed enhancement studies and was not intended for direct mechanistic comparison with Se soaking due to differences in treatment form, concentration scale, and application protocol. Seeds were placed in mesh bags and immersed in 500 mL of soaking solution, which was renewed daily. Seeds were incubated in an artificial climate chamber at 15 °C for 7 days until visible germination symptoms appeared, including embryo swelling and protrusion and endosperm softening. The extended soaking duration (7 days at 15 °C) was used to simulate early-season low-temperature conditions in cold-region direct-seeded rice systems, ensuring sufficient water uptake and uniform physiological activation prior to germination assessment. Germination incubation was conducted under a 12 h light/12 h dark photoperiod with relative humidity of 70–80%.

For the Se priming treatment, selected and air-dried rice seeds were completely submerged in a 50 mmol L^-^¹ sodium selenite solution and primed for 24 h at 25 °C in the dark. After priming, the seeds were dried in a forced-air drying oven at 25 °C until their weight returned to approximately the original level before treatment and were stored at 4 °C until use. After soaking and priming, the seeds were incubated in an artificial climate chamber at 20 °C for germination. A seed was considered germinated when the radicle visibly protruded. The number of germinated seeds was recorded every other day, and the final germination rate was determined on Day 9.

#### 2.2.2 Pot experiment

The pot experiment was conducted at Northeast Agricultural University, Harbin, Heilongjiang Province. Seeds subjected to the five treatments described above were sown on May 10. For each treatment, four pots were used as replicates, and each pot contained 60 seeds, which were evenly broadcast on the soil surface. The seeds were gently pressed so that approximately half of each seed was embedded in the soil, minimizing displacement. For each variety, 20 pots were used (5 treatments × 4 replicates), for a total of 40 pots across the two varieties. Seedling emergence was recorded 30 days after sowing, and the number of tillers was determined at the five-leaf stage.

#### 2.2.3 Field experiments

Based on the results of the germination and pot experiments, the combined treatment of prohexadione-calcium and selenium (PC+Se) resulted in the best performance and was therefore selected for field validation to confirm its effectiveness under realistic agricultural conditions. The experiment was conducted in 2021 at the Academy of Agricultural Sciences in Harbin, Heilongjiang Province, and included two treatments. Harbin has a humid temperate continental monsoon climate with a mean annual temperature of approximately 3–4 °C, an annual precipitation of approximately 550 mm, and a frost-free period of approximately 130 days. In the control (W) treatment, the seeds were soaked in water at 15 °C for 7 days after seed coating. In treatment 2, the coated seeds were immersed in a solution containing 0.1 mg L^-^¹ PC and 0.3 mg L^-^¹ Se at 15 °C for 7 days. The seeds were broadcast using unmanned aerial vehicle technology at a seeding rate of 50 kg ha^-^¹. The fertilization rates and application methods were identical across the treatments. The total nitrogen application rate was 100 kg ha^-^¹, while phosphorus and potassium were applied at rates of 75 kg P_2_O_5_ ha^-^¹ and 70 kg K_2_O ha^-^¹, respectively. Water management, weed control, and pest management followed local agronomic practices for direct-seeded rice. Of this, 65 kg ha^-^¹ of slow-release urea was applied as basal fertilizer, corresponding to 28.6 kg N ha^-^¹. Nitrogen fertilizer was applied at the basal, tillering, and panicle stages at proportions of 60%, 20%, and 20%, respectively. Phosphorus (P_2_O_5_) was applied at 75 kg ha^-^¹, with a basal-to-panicle ratio of 5:1, whereas potassium (K_2_O) was applied at 70 kg ha^-^¹ and equally divided between the basal and panicle stages.

Field experiments were conducted in 2022 at the Qianfeng and Erdaohe Farms in Jiansanjiang, Heilongjiang Province. At 15 °C, two seed-soaking treatments were applied, using either clear water or a PC+Se solution. After soaking, field sowing was carried out using unmanned aerial vehicle technology at a seeding rate of 75 kg ha^-^¹. The experimental design included four replicates per treatment. Nitrogen, phosphorus, and potassium were applied at 90, 75, and 70 kg ha^-^¹. The timing and proportions of nitrogen, phosphorus, and potassium application were consistent with those used above. In all the experiments, proper disease and weed control measures were implemented to avoid yield loss.

### 2.3 Determination items and methods

#### 2.3.1 Seed germination rate

One hundred seeds were used for the germination test. The seeds were considered germinated when the radicle protruded. The number of germinated seeds was recorded every other day for seven consecutive days, and the germination rate was calculated as the percentage of germinated seeds relative to the total number of tested seeds.

The germination potential (GP) was calculated as follows: *GP = n/N × 100%.*

where n represents the number of seeds that germinated within the first three days, and N represents the total number of seeds.

The germination index (GI) was calculated as follows: GI = Σ(Gt/Dt).

where G_t_ is the number of seeds that germinated on day D_t_ and D_t_ is the corresponding germination time (d).

#### 2.3.2 Determination of stress resistance indices

After the soaking and priming treatments, several physiological parameters were measured. The soluble protein content was determined using the Coomassie brilliant blue method (Bradford, 1976). Amylase activity was measured using the 3,5-dinitrosalicylic acid method (Miller, 1959), and MDA content was determined using the thiobarbituric acid method (Heath and Packer, 1968). The proline content was measured using the acid-ninhydrin method (Bates et al., 1973).

Superoxide dismutase (SOD) activity was determined using the nitroblue tetrazolium photoreduction method (Beauchamp and Fridovich, 1971). Peroxidase (POD) activity was measured using the guaiacol method (Chance and Maehly, 1955), and catalase (CAT) activity was determined by measuring ultraviolet absorption (Aebi, 1984).

#### 2.3.3 Emergence rate

In the pot experiment, the number of emerged seedlings was recorded 30 days after sowing, and the emergence rate was calculated as the percentage of emerged seedlings relative to the total number of sown seeds.

#### 2.3.4 Tiller number and dry weight

At the five-leaf stage, the number of tillers per plant was recorded. All the plants were then collected, washed, and separated into stems and leaves. The samples were heated to 105 °C to inactivate the enzyme and then dried to constant weight at 85 °C before the dry biomass (g) was determined.

#### 2.3.5 Rice yield and yield components

In the field experiment, a 0.16 m² area with uniform growth was sampled at maturity. The plants were separated into stems, leaves, and panicles. The number of panicles was recorded before threshing. After threshing, yield components, including grain number per panicle, the seed-setting rate, and the 1000-grain weight, were determined.

In addition, a 100 m² area was mechanically harvested, and the grain weight was measured after threshing. The grain yield was adjusted to a standard moisture content of 14.5%.

#### 2.3.6 Endogenous hormone measurement

Endogenous gibberellin (GA) and abscisic acid (ABA) contents of seeds from all four treatments (water [W], selenium [Se], prohexadione-calcium [PC], and combined PC+Se) were analyzed by a third-party laboratory (Scientific Dog Co., Hefei, Anhui, China). Seed samples were prepared according to the laboratory’s standard protocol, and hormone concentrations were quantified using targeted LC-MS. The specific GA species quantified included GA_1_, GA_3_, GA_4_, and GA^7^. Hormone sampling was conducted after 7 days of seed soaking under low-temperature conditions, and measurements were performed using whole seed tissues. Each treatment included three biological replicates. The laboratory provided raw LC-MS data and calculated hormone concentrations, which were subsequently used for statistical analysis.

### 2.4 Data analysis

All data were processed using Microsoft Excel 2019 and analyzed with SPSS 26.0. Treatment differences were examined using Duncan’s multiple-range test at the 0.05 significance level, with four biological replicates per treatment. The direct and indirect effects of variety and seed treatment on germination rate (GR) were analyzed by structural equation modeling (SEM) using the R lavaan package. The initial SEM variables were selected based on significant correlations with germination rate (P < 0.05) and their established physiological roles in antioxidant defense, osmotic regulation, reserve metabolism, and hormonal signaling under chilling stress. Model identification was ensured by confirming that the number of observed variances and covariances exceeded the number of estimated parameters, and the final model was over-identified (df > 0), allowing unique parameter estimation. No *post-hoc* model modifications were performed, and all structural paths were retained based on *a priori* hypotheses to avoid data-driven fitting. Model fit was evaluated based on χ²/df, CFI, TLI, RMSEA, and SRMR. Bootstrap resampling (5,000 iterations) with bias-corrected 95% confidence intervals was applied to test the stability of parameter estimates and indirect effects.

## 3 Results

### 3.1 Germination test

#### 3.1.1 Germination index

**Figure 2:**
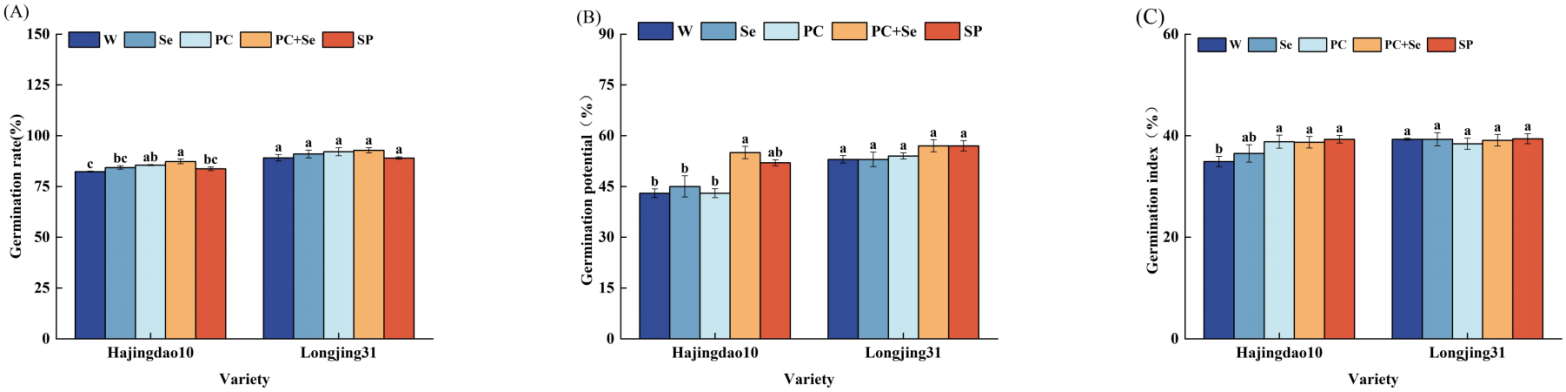
Effects of seed treatments on the germination rate **(A)**, germination potential **(B)**, and germination index **(C)** under low-temperature conditions. W, water soaking; Se, selenium soaking; PC, prohexadione-calcium soaking; PC+Se, combined prohexadione-calcium and selenium soaking; SP, selenium priming. The bars represent the means ± SEs (n = 4). Different lowercase letters indicate significant differences among treatments at P < 0.05 according to Duncan’s multiple range test.

As presented in **Table 2**, both seed treatment and variety strongly affected the GR and germination potential (P < 0.01), whereas neither factor significantly affected the germination index. No significant interaction between variety and seed treatment was detected. The seed treatment slightly increased the germination index of Longjing 31, but the difference was not statistically significant. For the low-temperature–sensitive variety Hajingdao 10, the seed treatments increased the GR and germination potential by 2.4–6.5% and 0–27.9%, respectively. In contrast, the germination index changed only slightly among the treatments and remained statistically unchanged. This suggests that seed treatments primarily enhanced the final germination capacity rather than significantly altering the speed or synchrony of germination under low-temperature conditions. Compared with the control treatment, the combined soaking treatment with PC+Se significantly increased the GR (**Figure 2**).

**Table 2:** Analysis of variation in the effects of variety and seed treatment on seed germination indices.

Factor	Germination rate	Germination potential	Germination index	Soluble protein	α-amylase	β-amylase	Total amylase
Variety	**	**	ns	*	**	**	**
Treatment	*	**	ns	**	**	**	**
Variety×Treatment	ns	ns	ns	ns	ns	**	ns

*, ** indicates significance at the 0.05 and 0.01 probability levels, respectively, while ns indicates no significant difference.

#### 3.1.2 Soluble protein content

**Figure 3:**
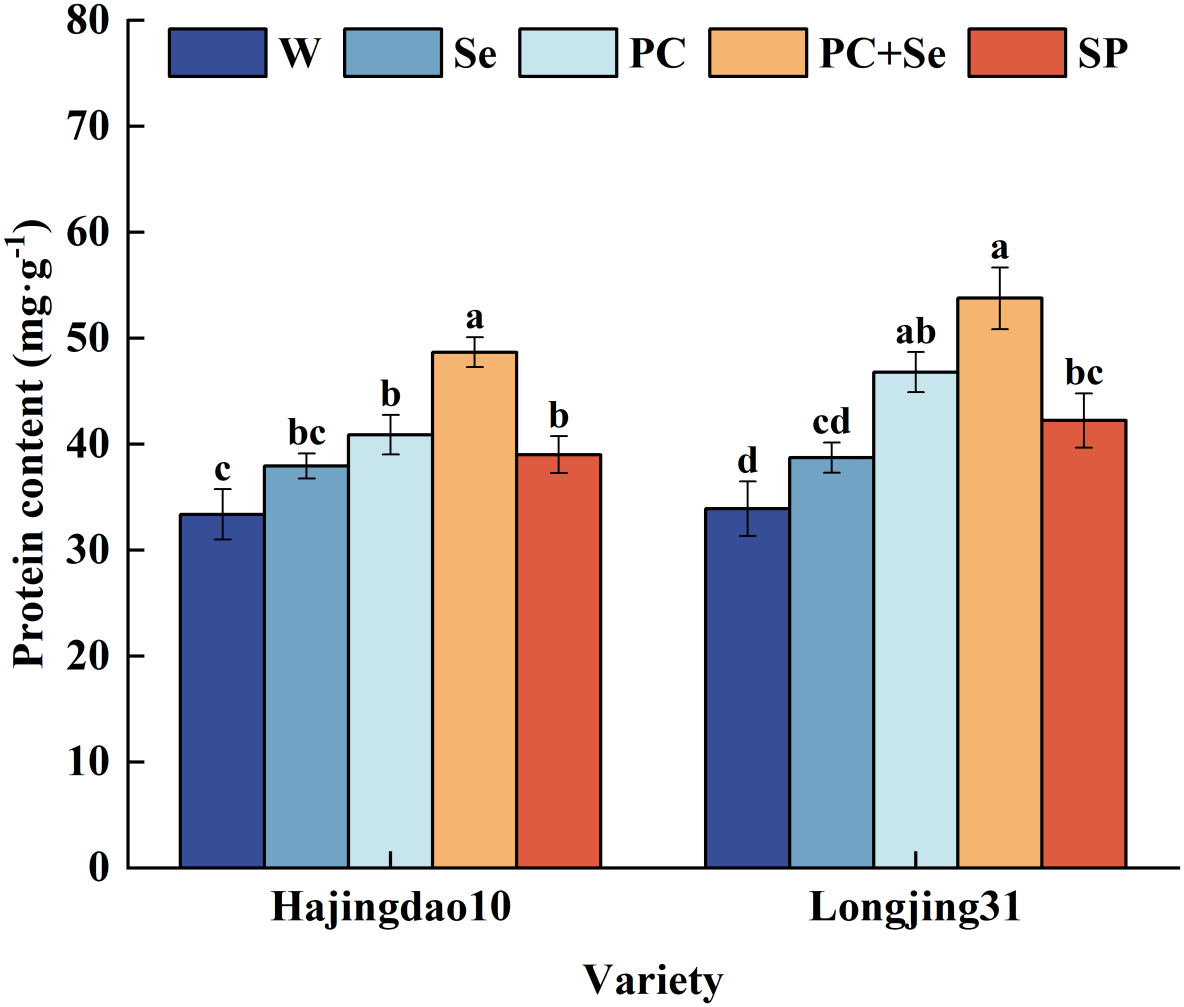
Effects of seed treatments on soluble protein content under low-temperature conditions. W, water soaking; Se, selenium soaking; PC, prohexadione-calcium soaking; PC+Se, combined prohexadione-calcium and selenium soaking; SP, selenium priming. The bars represent the means ± SEs (n = 4). Different lowercase letters indicate significant differences among treatments at P < 0.05 according to Duncan’s multiple range test.

As indicated in **Table 2**, seed treatment strongly affected the soluble protein content (P < 0.01). There was a significant difference in soluble protein content among the varieties (P < 0.05), but there was no significant interaction between seed treatment and variety. The seed treatment significantly increased the soluble protein content. Compared with the other treatments, the seed-soaking treatment with PC+Se resulted in the highest soluble protein content, reaching 48.67 mg g^-^¹ and 53.78 mg g^-^¹, respectively, which were significantly greater. The soluble protein content of ‘Longjing 31’ was, on average, 7.4% higher than that of Hajingdao 10 (**Figure 3**).

#### 3.1.3 Amylase activity

**Figure 4:**
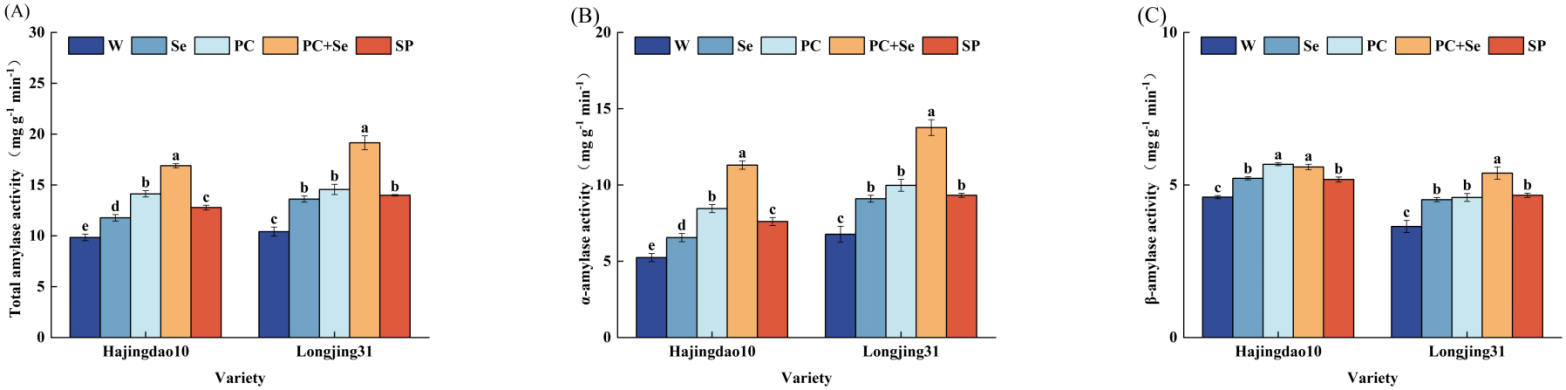
Effects of seed treatments on enzyme activity under low-temperature conditions. **(A)** Total amylase activity. **(B)** α-amylase activity. **(C)** β-amylase activity. W, water soaking; Se, selenium soaking; PC, prohexadione-calcium soaking; PC+Se, combined prohexadione-calcium and selenium soaking; SP, selenium priming. The bars represent the means ± SEs (n = 4). Different lowercase letters indicate significant differences among treatments at P < 0.05 according to Duncan’s multiple range test.

As shown in **Table 2**, both seed treatment and variety significantly affected total amylase, α-amylase, and β-amylase levels (P < 0.05). There was a highly significant interaction effect between seed treatment and variety on β-amylase activity (P < 0.01). The seed treatment increased amylase activity. Compared with those of the control, the activities of total amylase, α-amylase, and β-amylase increased by 30.8% to 84.0%, 25.0% to 103.3%, and 13.5% to 48.1%, respectively, after seed treatment. With the exception of the β-amylase activity of Hajingdao 10, the amylase activity in the PC+Se seed-soaking treatment was significantly greater than that in the other treatments (**Figure 4**). The activities of total-amylase, α-amylase, and β-amylase in ‘Longjing 31’ were higher than those in Hajingdao 10.

#### 3.1.4 MDA content

**Figure 5:**
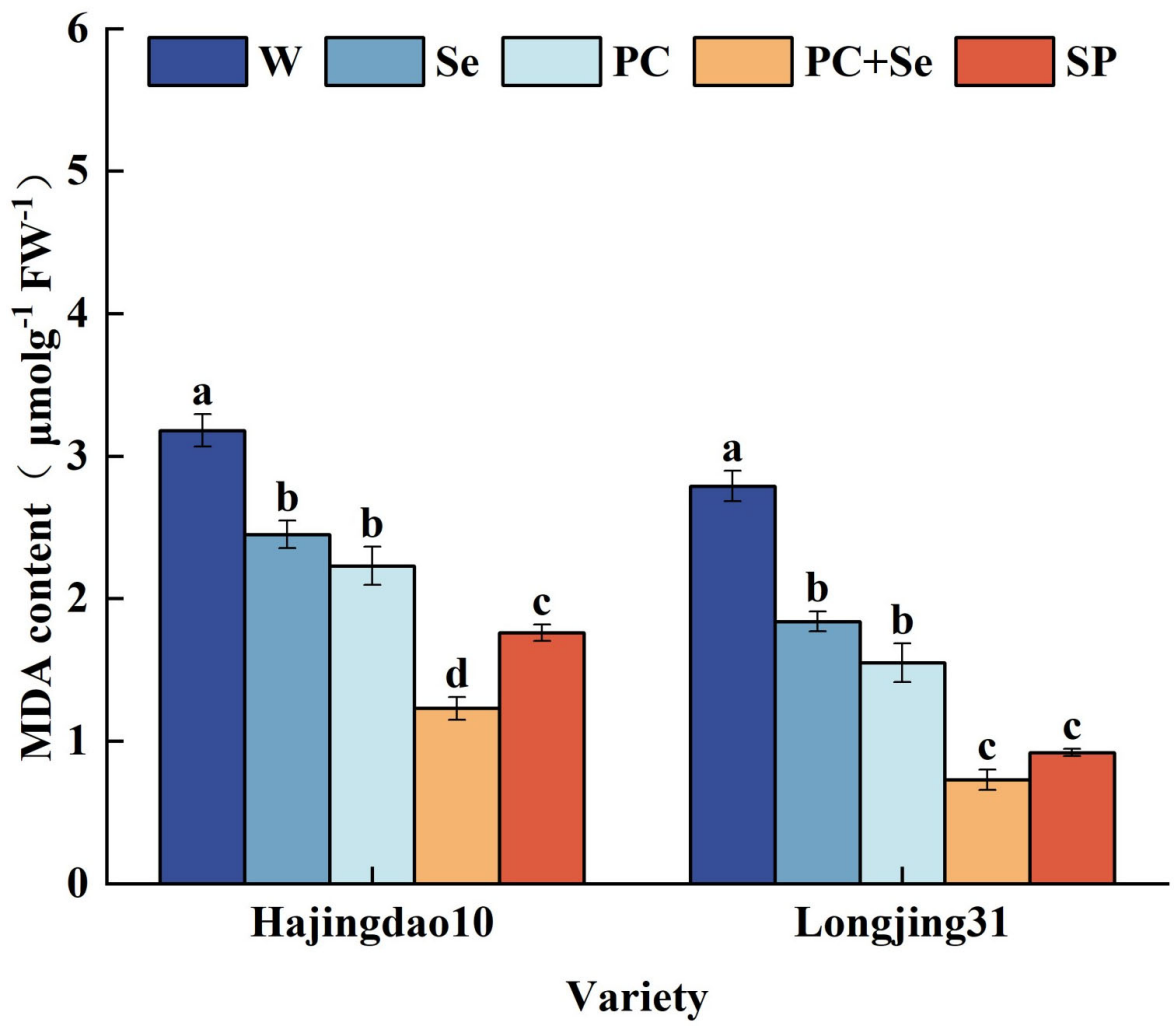
Effects of seed treatments on MDA content under low-temperature conditions. W, water soaking; Se, selenium soaking; PC, prohexadione-calcium soaking; PC+Se, combined prohexadione-calcium and selenium soaking; SP, selenium priming. The bars represent the means ± SEs (n = 4). Different lowercase letters indicate significant differences among treatments at P < 0.05 according to Duncan’s multiple range test.

As indicated by the data in **Table 3**, highly significant differences in the MDA content were detected between the seed treatments and varieties, but the interaction between them was not significant. The seed treatment significantly reduced the MDA content. Compared with that of the control, the MDA content decreased by 23.0-61.3% after seed treatment. The MDA content in the treated Longjing 31 variety was significantly lower than that in the Hajingdao 10 variety (**Figure 5**).

**Table 3:** Analysis of variance for the effects of cultivar and seed treatment on physiological indices under low temperature.

Factor	MDA	POD	SOD	CAT	Proline
Variety	**	**	**	**	**
Treatment	**	**	**	**	**
Variety×Treatment	ns	**	ns	ns	ns

*, ** indicates significance at the 0.05 and 0.01 probability levels, respectively, while ns indicates no significant difference.

#### 3.1.5 Antioxidant enzyme activity

**Figure 6:**
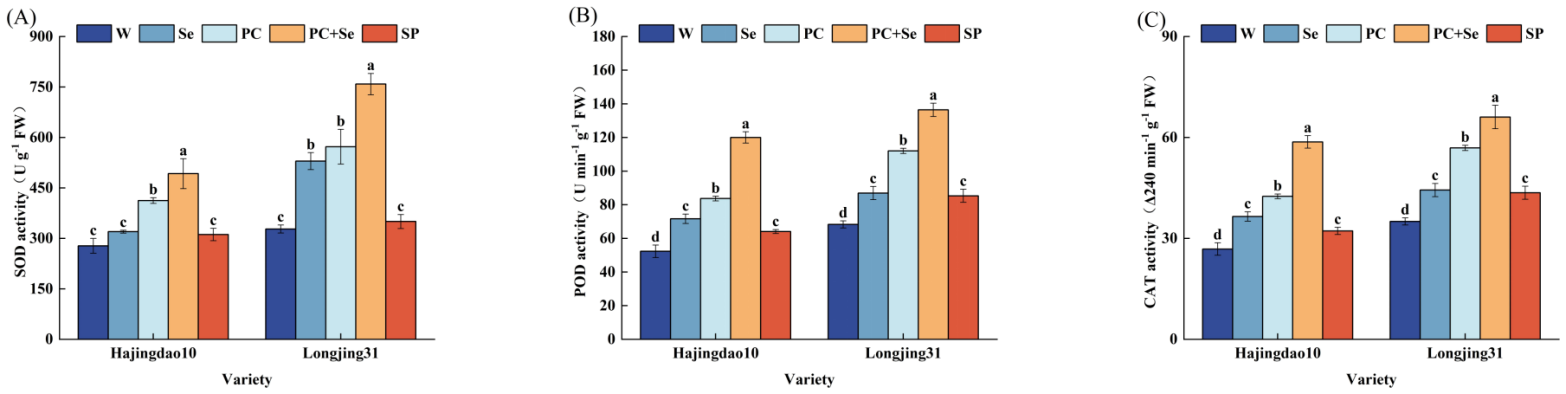
Effects of seed treatments on SOD **(A)**, POD **(B)**, and CAT activities **(C)**. W, water soaking; Se, selenium soaking; PC, prohexadione-calcium soaking; PC+Se, combined prohexadione-calcium and selenium soaking; SP, selenium priming. The bars represent the means ± SEs (n = 4). Different lowercase letters indicate significant differences among treatments at P < 0.05 according to Duncan’s multiple range test.

As shown in **Table 3**, highly significant differences in POD activity were detected among the seed treatments, varieties, and their interactions (P < 0.05). There were also highly significant differences in SOD activity and CAT activity between the different seed treatments and varieties (P < 0.05), but there was no significant interaction between them. The seed treatment increased the activities of antioxidant enzymes. Compared with those in the control treatment group, the activities of SOD, POD, and CAT in the treatment group increased by 6.9% to 131.5%, 22.5% to 129.3%, and 20.1% to 118.7%, respectively. Compared with those in the other treatments, the antioxidant enzyme activities in the PC+Se seed-soaking treatment were significantly greater (P < 0.05) (**Figure 6**). The antioxidant enzyme activities of ‘Longjing 31’ were higher than those of Hajingdao 10.

#### 3.1.6 Proline content

**Figure 7:**
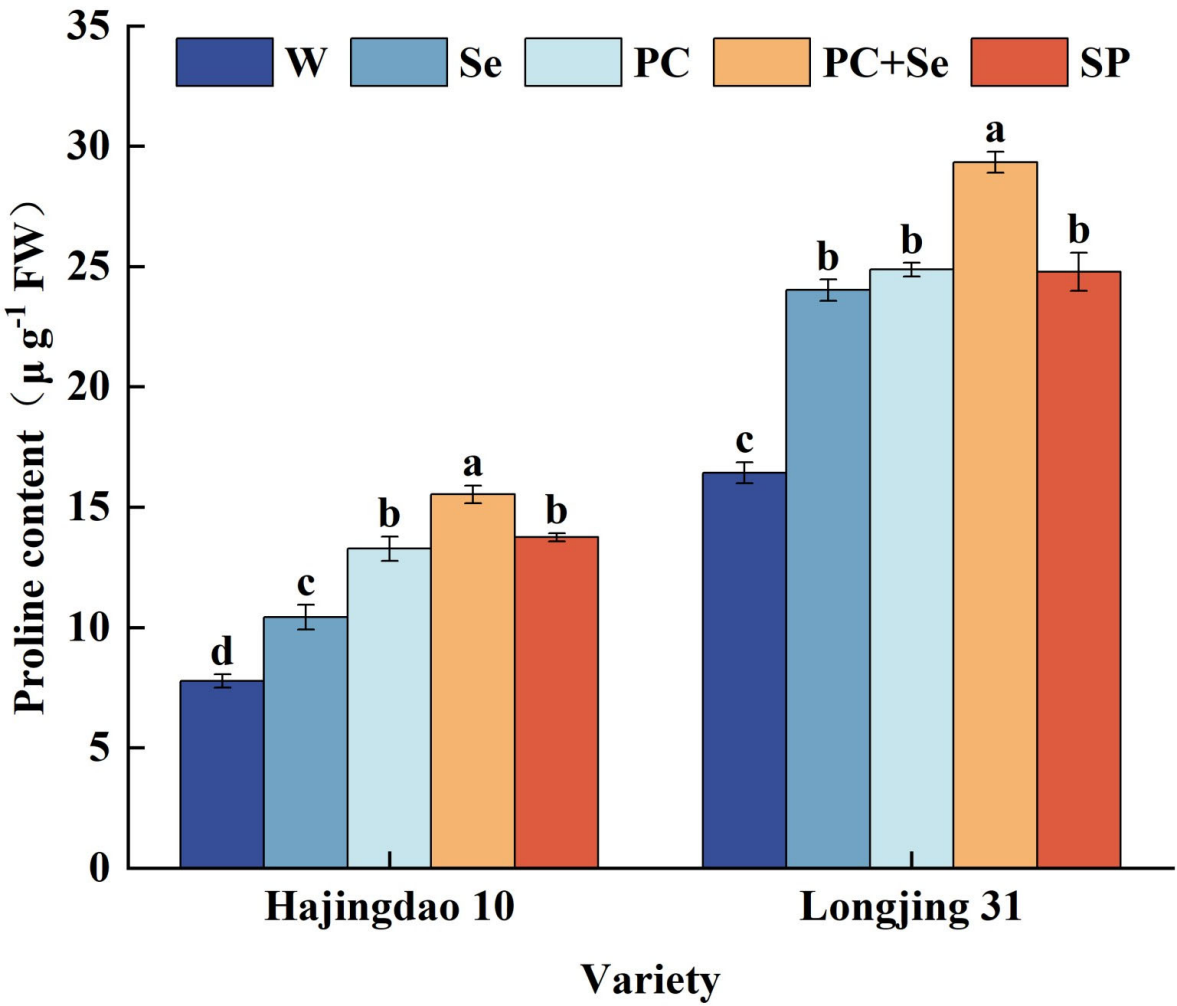
Effects of seed treatments on the proline content under low-temperature conditions. W, water soaking; Se, selenium soaking; PC, prohexadione-calcium soaking; PC+Se, combined prohexadione-calcium and selenium soaking; SP, selenium priming. The bars represent the means ± SEs (n = 4). Different lowercase letters indicate significant differences among treatments at P < 0.05 according to Duncan’s multiple range test.

As shown in **Table 3**, there were highly significant differences in the proline content between the seed treatments and varieties (P < 0.01), but no significant interaction was detected between them. The seed treatment significantly increased the proline content. Compared with that in the control treatment group, the proline content significantly increased by 34.1–99.7% in the seed treatment group (P < 0.05). The proline content of ‘Longjing 31’ was greater than that of Hajingdao 10, and the impact of seed treatment on the proline content of ‘Longjing 31’ was more significant (**Figure 7**).

### 3.2 Pot experiment

As shown in **Table 4**, variety did not significantly affect direct-seeded rice emergence but did affect tiller number and dry matter accumulation. The effects of the seed treatment on emergence, tiller number, and dry matter accumulation were highly significant (P < 0.01). Compared with those in the control treatment, the emergence rate, tiller number, and dry matter accumulation of plants in the PC+Se treatment increased by 8.22% to 10.29%, 13.44% to 24.38%, and 18.24% to 22.74%, respectively. In addition to the SP treatment, soaking seeds with Se or PC alone also significantly increased the emergence rate, tiller number, and dry matter accumulation (P < 0.05). The tiller number and dry weight of ‘Longjing 31’ were both greater than those of Hajingdao 10.

**Table 4:** Effects of seed treatment on emergence rate, tillering, and dry matter accumulation in the pot experiment.

Variety	Treatment	Seedling emergence rate (%)	Number of tillers (Plant pot^-1^)	Dry matter weight (g pot^-1^)
Hajingdao 10	W	73.18 bc	87 d	3.40 b
	Se	77.08 ab	102 b	3.80 a
	PC	77.08 ab	107 ab	3.77 a
	PC+Se	78.78 a	108 a	4.02 a
	SP	64.02 c	93 c	3.21 b
Longjing 31	W	68.33 b	83 b	2.99 b
	Se	73.65 a	92 a	3.40 a
	PC	74.58 a	92 a	3.47 a
	PC+Se	75.42 a	94 a	3.67 a
	SP	70.83 ab	83 b	3.08 b
	Variety	ns	**	**
	Treatment	**	**	**
	Variety*Treatment	**	*	ns

W, water soaking; Se, selenium soaking; PC, prohexadione-calcium soaking; PC+Se, combined soaking of prohexadione-calcium and selenium; SP, selenium priming. Values followed by different lowercase letters within a column indicate significant differences at P < 0.05 according to Duncan’s multiple range test. *P < 0.05; **P < 0.01; ns, not significant.

### 3.3 Field trials

As shown in **Table 5**, PC+Se seed soaking significantly increased seedling emergence rates by 4.7–9.4% across all three field site-years (P < 0.05). For yield components, the number of panicles per unit area showed a slight numerical increase in PC+Se plots, while grain number per panicle, seed-setting rate, and 1000-grain weight remained statistically similar between PC+Se and control treatments at all sites (**Table 5**). Grain yield showed a consistent positive numerical trend in PC+Se plots across all three site-years: +3.7% in 2021 Harbin (6.54 vs. 6.78 t ha^-^¹), +3.3% in 2022 Erdaohe (8.36 vs. 8.64 t ha^-^¹), and +3.5% in 2022 Qianfeng (9.35 vs. 9.68 t ha^-^¹). However, these yield differences did not reach statistical significance (P > 0.05) at any site. The consistent directional trend in yield improvement across three independent site-years, combined with statistically significant improvements in emergence rate, suggests agronomic potential that warrants further investigation. Future field trials with larger plot sizes (>500 m²), multi-year replication across diverse environments, and dose optimization are recommended to determine whether the yield benefits observed here can be statistically confirmed under commercial production conditions.

**Table 5:** Effects of seed treatment on yield and yield composition in field trials.

Time	Site	Treatment	Emergence rate %	Spike number m^-2^	Kernels per spike Per	Seed setting rate %	1000-grain weight g	Grain yield t/ha
2021	Harbin	CK	38.50 ± 1.0 b	211 ± 10a	164 ± 6.2a	85.39 ± 0.02a	31.20 ± 0.07a	6.54 ± 0.35a
		PC+Se	43.20 ± 0.4 a	213 ± 2a	166 ± 6.5a	85.71 ± 0.01a	31.51 ± 0.35a	6.78 ± 0.2a
2022	Erdaohe	CK	31.17 ± 0.8 b	475 ± 9.3a	70 ± 0.4a	91.5 ± 0.43a	27.15 ± 0.06a	8.36 ± 0.18a
		PC+Se	40.58 ± 0.8 a	490 ± 8.7a	71 ± 0.9a	92.1 ± 1.22a	27.34 ± 0.09a	8.64 ± 0.43a
	Qianfeng	CK	56.70 ± 1.0 b	541 ± 4.1a	65 ± 0.71a	93.1 ± 0.55a	28.42 ± 0.04a	9.35 ± 0.11a
		PC+Se	60.24 ± 0.6 a	553 ± 15a	66 ± 0.71a	93.2 ± 0.66a	28.5 ± 0.07a	9.68 ± 0.27a

PC+Se, combined soaking; CK, control (water soaking). Values followed by different lowercase letters within a column indicate significant differences at P < 0.05 according to Duncan’s multiple range test.

### 3.4 Structural equation modeling

**Figure 8:**
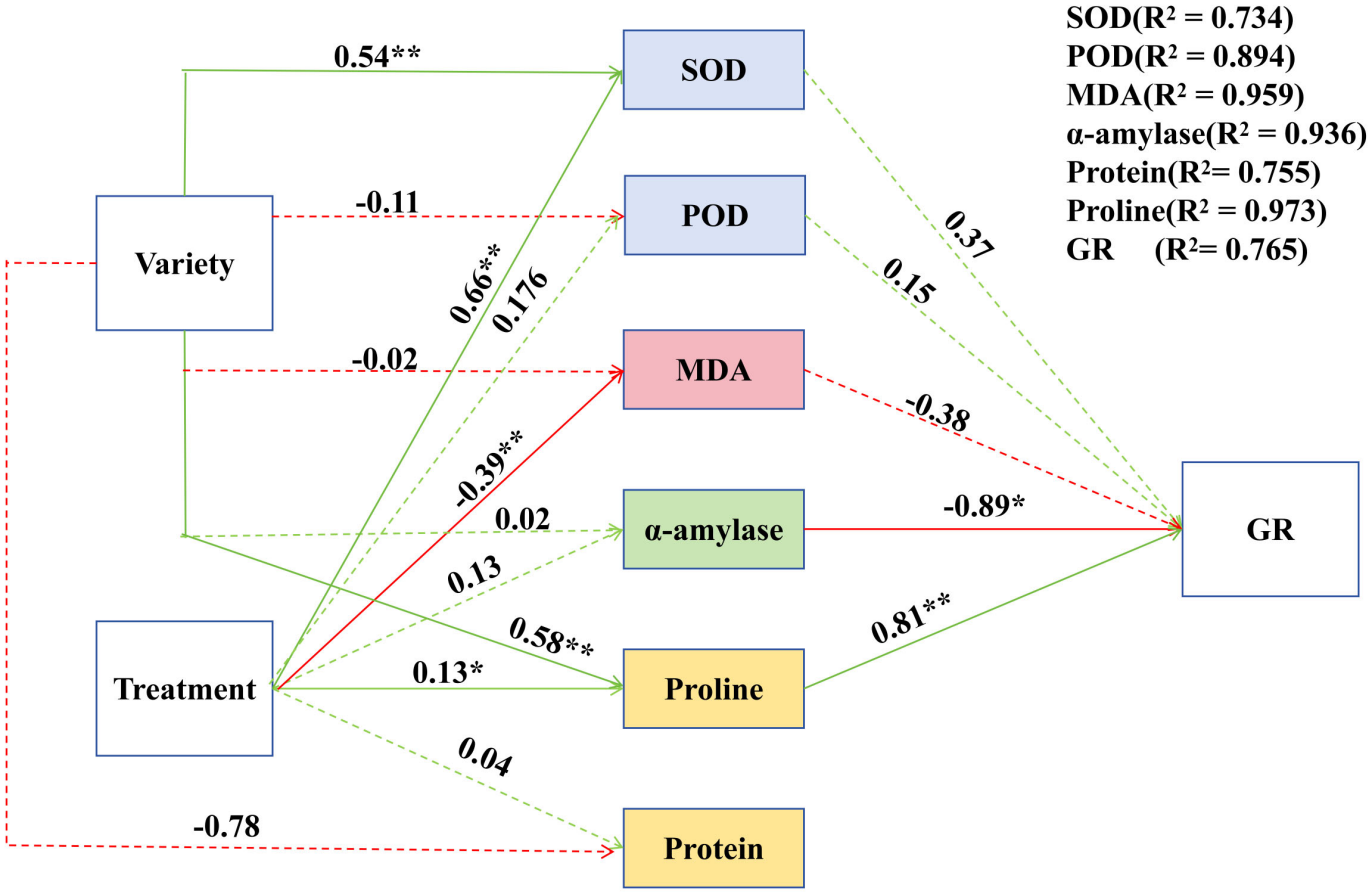
Structural equation model (SEM) illustrating direct and indirect effects of seed treatment and variety on germination rate (GR) under low-temperature stress. Green arrows indicate positive effects, while red arrows indicate negative effects. Solid lines represent significant paths (P < 0.05), whereas dashed lines indicate non-significant paths. Standardized path coefficients (β) are shown next to each arrow, with significance levels denoted as *P < 0.05 and **P < 0.01. Model estimation was performed using maximum likelihood in the R package *lavaan*. Model fit was evaluated using multiple indices, including χ²/df, CFI, TLI, RMSEA, and SRMR, showing acceptable overall fit (χ²/df = 1.99, CFI = 0.978, TLI = 0.902, RMSEA = 0.203, SRMR = 0.020). Proportion of variance explained (R²): GR = 0.765; MDA = 0.959; Proline = 0.973; α-amylase = 0.936; SOD = 0.734; soluble protein = 0.755; POD = 0.894. SOD, superoxide dismutase; POD, peroxidase; MDA, malondialdehyde; GR, germination rate.

As shown in **Figure 8**, structural equation modeling (SEM) was used to quantify the direct and indirect effects of physiological traits on germination rate (GR) under chilling stress. The model explained a substantial proportion of variance in GR, with a coefficient of determination (R²) of 0.765. Standardized path coefficients indicated that proline had a significant positive effect on GR (β = 0.81, P < 0.01), whereas α-amylase showed a significant negative effect (β = -0.89, P < 0.05). In contrast, SOD (β = 0.37, ns), POD (β = 0.15, ns), and MDA (β = -0.38, ns) showed no significant direct effects on GR. The model further explained variance in all endogenous variables, with R² values of 0.734 for SOD, 0.894 for POD, 0.959 for MDA, 0.936 for α-amylase, 0.755 for protein, 0.973 for proline, and 0.765 for GR.

## 4 Discussion

### 4.1 Rice seed germination and emergence

The observed discrepancy between germination rate and germination index suggests that seed treatments primarily improved seed viability under low-temperature stress, while having limited effects on the temporal dynamics of germination. This indicates that physiological recovery processes, rather than germination synchrony or speed regulation, were the main limiting factor under chilling conditions. The germination rates (GRs) of seeds generally increase initially but decrease beyond a suitable temperature range. In this study, after 7 days of low-temperature treatment (15 °C), only the PC+Se combination significantly promoted germination of the cold-sensitive varieties under 20 °C in an artificial climate. Seeds exposed to low temperature were grown in pots at 15–18 °C. Se, PC, and the PC+Se combination significantly increased the emergence rate of both cold-sensitive and cold-tolerant varieties. The combination of PC+Se had a more significant effect on promoting rice tillering in a pot experiment and increased rice yield in a field experiment. These experimental results indicate that the harm to seed growth caused by short-term low-temperature exposure may be temporary and that the damage can automatically recover when the temperature conditions become suitable. The observation that differences among treatments decreased when seeds were transferred from 15 °C to 20 °C for germination suggests that treatment effects on cold tolerance are most pronounced under sustained low-temperature conditions, consistent with the pot experiment findings (**Table 4**). This has practical implications for germination test design: future studies evaluating seed treatment efficacy for cold stress should consider conducting germination tests at temperatures more representative of actual field conditions during sowing (e.g., 13-18 °C in Heilongjiang Province) rather than at standard laboratory germination temperatures (20-25 °C), which may underestimate treatment benefits. However, standardized germination tests use defined temperatures for reproducibility; modifications should be clearly specified and justified in each study. Previous studies have shown that both Se soaking and SP can significantly promote seed germination. In contrast, in this study, only the GR and rice growth improved considerably after Se soaking. The reasons why Se does not promote seed germination remain unclear. The germination rate of ‘Longjing 31’ was greater than that of Hajingdao 10 in the germination test. Nevertheless, there was no significant difference in the number of seedlings between the two groups in the pot experiment. However, the number of tillers and dry weight of Hajingdao 10 were significantly greater than those of ‘Longjing 31’. Studies have shown that it is necessary to form strong seedlings and achieve vigorous early-stage growth (Fauzi et al., 2021; Meng et al., 2018). This may explain the higher tillering and dry weight of Hajingdao 10.

### 4.2 Physiological mechanism by which seed treatment promotes rice germination

Differences in cold tolerance among rice varieties during germination and seedling emergence vary significantly. These differences are mainly due to antioxidant defense capacity, hormone regulation, amylase activity, protein synthesis, and amino acid metabolism. In this study, the cold-tolerant variety Longjing 31 showed higher antioxidant enzyme activity and amylase levels. It also exhibited higher proline and soluble protein contents, along with lower MDA content (**Table 2**). SEM analysis of the varieties revealed that the path coefficient for antioxidant enzyme activity and proline content was positive, whereas the path coefficient for MDA was negative, and soluble protein was not significant. However, α-amylase activity showed a significant negative effect on GR (β = -0.89, P < 0.05), indicating that the role of α-amylase in cold tolerance is context-dependent and not simply positively associated with germination performance. The accumulation of MDA was significantly inhibited in low-temperature–resistant varieties, which was associated with higher antioxidant enzyme activity and increased levels of osmotic-responsive substances, with osmotic regulation likely playing a more direct role in maintaining cellular homeostasis and membrane stability. The results of this study are essentially consistent with those of previous studies. Previous studies have shown that antioxidant enzyme activities generally increase in rice genotypes under low-temperature stress, particularly superoxide dismutase (SOD), and that reduced MDA accumulation is associated with enhanced cellular protection and stress mitigation capacity (Hasanuzzaman et al., 2021), indicating that the antioxidant system primarily functions as a physiological buffering mechanism under chilling stress. In addition, different rice varieties present significant differences in levels of MDA, proline, soluble protein, and other indicators after low-temperature treatment; cold-resistant varieties present higher proline and soluble protein contents, which are accompanied by a trend toward increased antioxidant enzyme activity (Guo et al., 2022). The α-amylase activity and soluble protein activity of the resistant varieties in this study were also high. However, the path coefficient in the SEM analysis was not significant, indicating that soluble protein levels are not decisive factors in variety resistance. The osmotic regulation capacity of varieties, particularly proline accumulation, may be more critical for cold tolerance during germination, which is consistent with previous studies (Lutts et al., 2016). However, studies have also shown that α-amylase activity is a core indicator of cold tolerance during rice germination (Kim et al., 2026). To reconcile these apparently inconsistent observations and to better interpret varietal differences, the SEM results provide mechanistic insight into the physiological basis of cold tolerance, particularly by clarifying the net effects of α-amylase after accounting for antioxidant and osmotic regulation pathways.

The SEM path analysis provides important mechanistic context for variety differences. The significant positive direct effect of proline indicates that the intrinsic cold tolerance advantage of Longjing 31 is primarily associated with enhanced osmotic regulation capacity. In contrast, antioxidant enzyme activity (SOD) showed a positive but non-significant association with variety, suggesting that antioxidant responses play a secondary, buffering role rather than acting as a primary determinant of germination performance. Accordingly, breeding for cold tolerance in direct-seeded rice may benefit from considering antioxidant enzyme activity as a supporting selection trait rather than α-amylase activity, which showed a significant negative association with germination performance. Varieties with high baseline SOD activities may also potentially respond more strongly to exogenous antioxidant-enhancing treatments like PC+Se soaking, suggesting a genotype × treatment interaction effect that could be exploited in crop management strategies. The non-significant variety effect on emergence in the pot experiment (**Table 4**), despite significant germination differences in controlled conditions, further suggests that the variety advantage is temperature-dependent, manifesting most clearly under the most extreme chilling conditions of the controlled germination test.

### 4.3 Physiological mechanism through which seed soaking promotes rice germination

Seed soaking treatments significantly enhanced germination performance under chilling stress by modulating a coordinated physiological network. In this study, no significant effects of seed treatments on endogenous hormone levels (GA and ABA) were observed (**Supplementary Table 1**), suggesting that hormonal changes were not the dominant drivers of the observed germination responses under the experimental conditions. The lack of significant differences in endogenous GA levels following PC treatment may be attributed to the physiological state of seeds during early imbibition under low-temperature conditions, when GA biosynthesis and signaling are not fully stabilized. Although GA metabolism is expected to be inhibited by prohexadione-calcium, the response appears to be isoform-specific rather than uniform across all GA components. In particular, different GA forms (GA1, GA3, GA4, and GA7) exhibited distinct response patterns, suggesting hormonal redistribution rather than a consistent change in total GA content. In addition, GA signaling regulation is highly spatially and temporally dynamic, and whole-seed measurements may not fully capture localized regulatory effects. Therefore, the observed lack of significant change in total GA likely reflects the integration of divergent isoform-level responses rather than the absence of PC effects.

Structural equation modeling (SEM) provided an integrated view of these relationships (**Figure 8**), demonstrating that germination rate (GR) was primarily driven by osmotic regulation, with proline exerting the strongest direct positive effect (β = 0.81, P < 0.01), indicating its central role in maintaining cellular homeostasis during early germination under low-temperature conditions (Apel and Hirt, 2004; Mittler, 2017). In contrast, α-amylase activity showed a significant negative direct effect on GR (β = -0.89, P < 0.05), indicating that under chilling stress, its role is decoupled from germination promotion. The negative effect suggests that excessive starch hydrolysis may lead to an imbalance between sugar production and utilization, where hydrolytic products are preferentially redirected toward maintenance metabolism rather than growth, consistent with stress-induced metabolic reprogramming during germination (Bewley et al., 2013; Finch-Savage and Leubner-Metzger, 2006).

Antioxidant-related processes (SOD, POD) and lipid peroxidation (MDA) did not show significant direct effects on GR, but reflected upstream physiological stress status. Specifically, antioxidant enzymes were associated with stress regulation, while MDA indicated membrane damage under chilling stress. SEM results therefore indicate a hierarchical regulatory pattern in which osmotic adjustment is the primary determinant of germination, carbohydrate metabolism exhibits stress-dependent inhibition, and antioxidant processes function mainly as stress-response indicators.

In summary, combined PC+Se seed soaking at 0.1 mg L^-^¹ PC and 0.3 mg L^-^¹ Se significantly improved germination rate, seedling emergence, antioxidant enzyme activities (SOD, POD, CAT), proline content, and soluble protein accumulation under chilling stress conditions in direct-seeded rice. Structural equation modeling identified proline accumulation as the strongest positive direct contributor to low-temperature germination, while α-amylase showed a significant negative direct effect, interpreted as a stress-related response under conditions where metabolic constraints limit growth performance. Antioxidant enzyme activities (SOD, POD) contributed indirectly to germination performance by reflecting physiological stress status. Grain yield showed a consistent positive numerical trend across three field site-years (+3.3 to +3.7%) but these differences did not reach statistical significance, warranting further field validation with larger plot sizes and multi-year replication. No significant changes in endogenous GA or ABA content were observed, suggesting that hormonal changes were not the dominant drivers of germination regulation under the experimental conditions. These findings establish a mechanistic framework emphasizing osmotic regulation and stress metabolism over starch mobilization and hormonal regulation as primary determinants in low-temperature germination, and support PC+Se seed soaking as a practical, agronomically applicable strategy for improving seedling establishment stability in direct-seeded rice under early spring chilling conditions. Future research should investigate: (1) the molecular targets of PC and Se in activating proline gene expression during seed soaking; (2) genotype × treatment interactions across diverse rice varieties with characterized physiological profiles; (3) dose optimization for different regional temperature conditions; and (4) multi-year, multi-location field trials to confirm the agronomic value of this seed treatment strategy.

## Data Availability

The raw data supporting the conclusions of this article will be made available by the authors, without undue reservation.
