# Supplementary material for: Structural equation modeling reveals proline as the dominant pathway governing rice seed germination under chilling stress
Source: Front Plant Sci. 2026 Jul 13;17:1889530. doi: 10.3389/fpls.2026.1889530 (PMC13403325; doi:10.3389/fpls.2026.1889530)
Supplement: Supplementary file 1 [file Table1.doc]

| **Variety** | **Treatment** | **Replicates (n)** | **ABA** | **GA3** | **GA1** | **GA4** | **GA7** |
| --- | --- | --- | --- | --- | --- | --- | --- |
| Hajingdao 10 | W | 3 | 2795.33 ± 6.70 | 2541.72 ± 22.96 | 7415.14 ± 619.77 | 30709.16 ± 1851.34 | 2248.90 ± 677.96 |
| Hajingdao 10 | PC | 3 | 3375.17 ± 14.58 | 2095.88 ± 37.21 | 3209.74 ± 1651.44 | 25018.41 ± 10354.26 | 3425.74 ± 106.27 |
| Hajingdao 10 | PC+Se | 3 | 3138.96 ± 19.55 | 2065.83 ± 43.48 | 4706.05 ± 1321.82 | 23821.72 ± 875.56 | 3274.03 ± 82.27 |
| Hajingdao 10 | SP | 3 | 4100.39 ± 56.38 | 1617.91 ± 7.74 | 7242.15 ± 3007.44 | 3529.55 ± 583.44 | 3285.57 ± 86.20 |
| Hajingdao 10 | Se | 3 | 3923.69 ± 5.90 | 2693.14 ± 53.52 | 4050.44 ± 1808.71 | 30424.07 ± 2984.92 | 3399.82 ± 151.13 |
| Longjing 31 | W | 3 | 3248.77 ± 65.31 | 2247.24 ± 13.83 | 7788.84 ± 1683.34 | 23342.17 ± 629.86 | 3607.23 ± 102.29 |
| Longjing 31 | PC | 3 | 3390.80 ± 2.23 | 2116.52 ± 62.51 | 6366.42 ± 684.88 | 24174.76 ± 1604.34 | 3230.98 ± 261.50 |
| Longjing 31 | PC+Se | 3 | 2877.79 ± 2.51 | 2429.53 ± 23.07 | 7086.13 ± 3372.64 | 28328.08 ± 4429.91 | 3427.45 ± 80.54 |
| Longjing 31 | SP | 3 | 4137.19 ± 10.97 | 1873.53 ± 7.65 | 9300.60 ± 424.36 | 2742.07 ± 53.52 | 3151.53 ± 27.95 |
| Longjing 31 | Se | 3 | 3010.74 ± 28.66 | 2291.53 ± 31.49 | 4686.16 ± 1613.80 | 23824.18 ± 841.44 | 3604.19 ± 103.13 |

**Supplementary Table S1 Endogenous hormone contents in rice seeds under different seed treatments. Values are mean ± SE (n = 3).**

Notes: Values are mean ± SE (n = 3). Unit: pg g⁻¹ FW.
W, water soaking; Se, selenium soaking; PC, prohexadione-calcium soaking; PC+Se, combined soaking; SP, selenium priming.
